# Novel 14q32.2 paternal deletion encompassing the whole *DLK1* gene associated with Temple syndrome

**DOI:** 10.1186/s13148-024-01652-8

**Published:** 2024-05-07

**Authors:** Neus Baena, David Monk, Cinthia Aguilera, Mario F. Fraga, Agustín F. Fernández, Elisabeth Gabau, Raquel Corripio, Nuria Capdevila, Juan Pablo Trujillo, Anna Ruiz, Miriam Guitart

**Affiliations:** 1 Genetics Laboratory, Centre de Medicina Genòmica, Parc Taulí Hospital Universitari, Institut d’Investigació i Innovació Parc Taulí I3PT, Universitat Autònoma de Barcelona, Sabadell, Spain; 2 Institut d’Investigació Biomèdica de Bellvitge (IDIBELL), Hospital Duran i Reynals, L’Hospitalet de Llobregat, Barcelona, Spain; 3 University of East Anglia, Norwich Research Park, Norwich, NR4 7TJ UK; 4 Cancer Epigenetics and Nanomedicine Laboratory, Nanomaterials and Nanotechnology Research Center (CINN-CSIC), Health Research Institute of Asturias (ISPA), Institute of Oncology of Asturias (IUOPA) and Department of Organisms and Systems Biology (B.O.S.), University of Oviedo, Oviedo, Spain; 5 Rare Diseases CIBER (CIBERER) of the Carlos III Health Institute (ISCIII), Madrid, Spain; 6 Paediatric Endocrinology Department, Parc Tauli Hospital Universitari, Institut d’Investigació i Innovació Parc Taulí I3PT, Universitat Autònoma de Barcelona, Sabadell, Spain

**Keywords:** Temple syndrome (TS14), Methylation, *DLK1*, Deletion, DMR

## Abstract

**Background:**

Temple syndrome (TS14) is a rare imprinting disorder caused by maternal UPD14, imprinting defects or paternal microdeletions which lead to an increase in the maternal expressed genes and a silencing the paternally expressed genes in the 14q32 imprinted domain. Classical TS14 phenotypic features include pre- and postnatal short stature, small hands and feet, muscular hypotonia, motor delay, feeding difficulties, weight gain, premature puberty along and precocious puberty.

**Methods:**

An exon array comparative genomic hybridization was performed on a patient affected by psychomotor and language delay, muscular hypotonia, relative macrocephaly, and small hand and feet at two years old. At 6 years of age, the proband presented with precocious thelarche. Genes dosage and methylation within the 14q32 region were analyzed by MS-MLPA. Bisulfite PCR and pyrosequencing were employed to quantification methylation at the four known imprinted differentially methylated regions (DMR) within the 14q32 domain: *DLK1* DMR, IG-DMR, *MEG3* DMR and *MEG8* DMR.

**Results:**

The patient had inherited a 69 Kb deletion, encompassing the entire *DLK1* gene, on the paternal allele. Relative hypermethylation of the two maternally methylated intervals, *DLK1* and *MEG8* DMRs, was observed along with normal methylation level at IG-DMR and *MEG3* DMR, resulting in a phenotype consistent with TS14. Additional family members with the deletion showed modest methylation changes at both the *DLK1* and *MEG8* DMRs consistent with parental transmission.

**Conclusion:**

We describe a girl with clinical presentation suggestive of Temple syndrome resulting from a small paternal 14q32 deletion that led to *DLK1* whole-gene deletion, as well as hypermethylation of the maternally methylated DLK1-DMR.

**Supplementary Information:**

The online version contains supplementary material available at 10.1186/s13148-024-01652-8.

## Background

Temple syndrome (TS14, OMIM #616222) was described more than 30 years ago in a patient with a maternal uniparental disomy 14 (UPD(14)mat) due to a Robertsonian translocation (13;14) [1]. Classical TS14 phenotypic features include pre- and postnatal short stature, small hands and feet, muscular hypotonia, motor delay, feeding difficulties, weight gain, and precocious puberty [2, 3]. Clinical review of published cases showed that most patients, especially in infancy, were initially suspected to have Prader-Willi syndrome (PWS) or Silver-Russell syndrome (SRS), which is explained by the phenotypic overlap between TS14 and PWS (growth failure, hypotonia, and small hands and feet) and between TS14 and SRS (growth failure and feeding difficulties) [4–7].

TS14 is a rare imprinting disorder that is caused by genetic and epigenetic alterations including UPD(14)mat, imprinting defects and paternal microdeletions which lead to maternalization (i.e. the paternally derived chr14 resembles the maternal inherited copy) at the imprinted domain on chromosome 14q32 [3]. The 14q32 region harbors the paternally expressed genes *DLK1* and *RTL1* as well as the maternally expressed genes *MEG3* (also known as *GTL2*)*, RTL1as*, *MEG8* and large sno- and microRNA gene clusters [8–10].

The parent-of-origin-specific expression is regulated by two differentially methylated regions (DMRs) acting as imprinting control centers, the germline primary IG-DMR (*MEG3/DLK1*:IG-DMR; following the nomenclature recommended in Monk et al.[11]) located between the *DLK1* and *MEG3* gene and the somatic secondary *MEG3*-DMR (*MEG3*:TSS-DMR) overlapping the *MEG3* promotor region [2]. The germline-derived IG-DMR acts upstream of the secondary-derived somatic *MEG3* DMR and governs it in a hierarchical fashion [12, 13]. Two somatically acquired DMRs methylated on the maternal allele have recently been described, the *MEG8* DMR (*MEG8*: Int2-DMR) located in the intron 2 of the *MEG8* gene [14, 15] and the *DLK1* DMR (*DLK1*:Int1-DMR) overlapping the second exon of the *DLK1* gene [16]. The function of these two DMRs is currently unknown.

Previously, patients with TS14 have shown hypomethylation at the IG-DMR and *MEG3* DMR and hypermethylation at the *MEG8* DMR with an increased expression of the maternally expressed transcripts and silencing of paternally expressed genes. Currently, 128 patients with TS14 have been described in the literature allowing the frequency of the underlying molecular causes to be defined. The majority present with UDP(14)mat (57%), epigenetic defects (31%) and microdeletions (12%) [3, 7, 17–22]. The size of microdeletions vary in length with the majority extending for ~ 1 Mb encompassing the *EML1*, *EVL*, *YY1, SCL25A29, SLC325A47, BEGAIN, DLK1, MEG3, RTL1* genes, as well as the sno- and microRNA clusters, among others [18, 23].

Here, we report a family with a ~ 69 Kb microdeletion that led to a whole-gene deletion of *DLK1*. Paternal transmission resulted in clinical features consistent with Temple syndrome. The proband exhibited relative hypermethylation at the *DLK1* and *MEG8* DMR and normal methylation at the IG-DMR and *MEG3*-DMR.

## Results

The proband is the first daughter of non‐consanguineous parents and was born at 42 weeks following an uncomplicated pregnancy with a weight of 2900 g (p7,− 1.6SD), length of 50 cm (0 SDS) and head circumference at birth of 34 cm (+ 1SD), which increased when the patient was one month old (+ 2SD), consistent with relative macrocephaly at birth. She presented stereotypies at 6 months old, and an electroencephalogram was performed with a normal result. She was followed up at the Childhood Development and Early Intervention Centre (CDIAP) due to muscular hypotonia. Autonomous sitting was done at 18 months old and she was able to walk independently at 21 months.

**Fig. 1:**
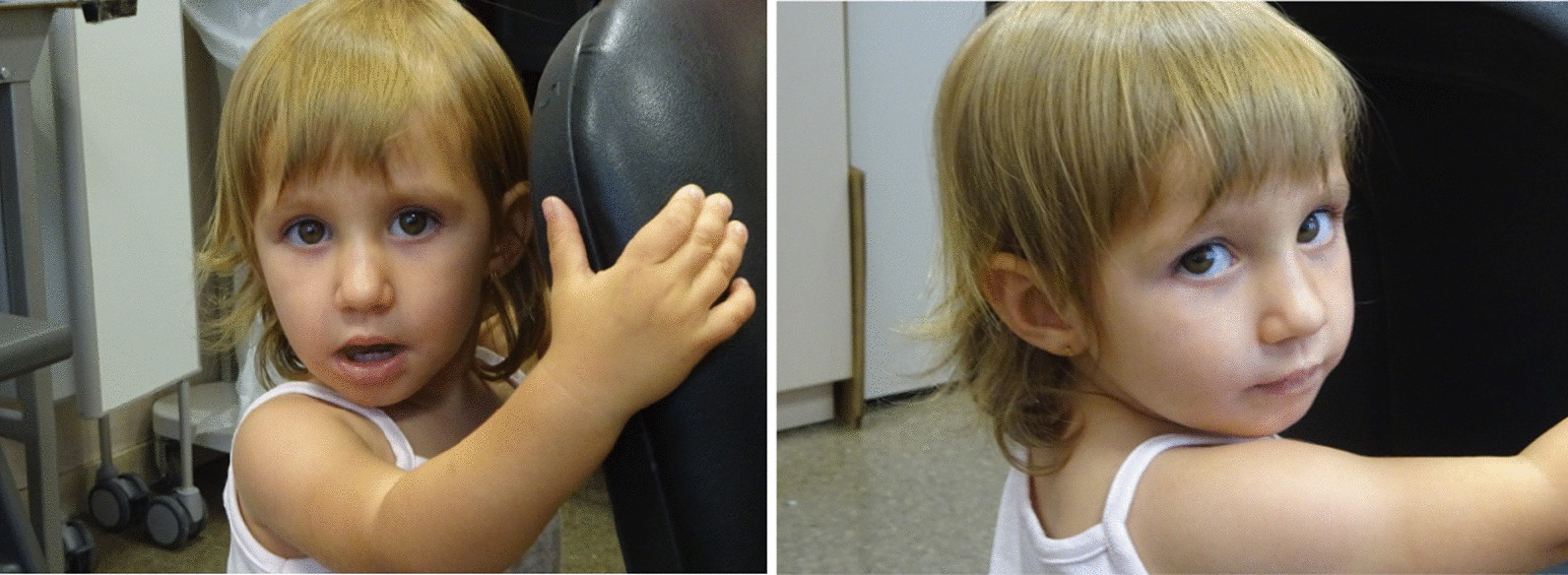
Clinical traits present in our case. The photographs show prominent forehead, almond-shaped eyes, broad nasal tip small hand and attached lobe in ear

The proband was referred to the Clinical Genetic Unit at 2 years old due to psychomotor and language delay. The patient also presented with growth failure (height 82.7 cm, − 2.0 SDS) and muscular hypotonia. At 4 years old, a clinical reassessment noticed several other clinical features resembling Temple syndrome (Table 1), such as prominent forehead, almond-shaped eyes, a broad nasal tip, ears with attached lobes, hyperextensible joints, small hands and mild intellectual disability (Fig. 1). She manifested shyness and motor clumsiness and attended a school with educational support. At 5 years, an umbilical hernia was repaired. Follow-up at the endocrinologist unit evidenced thelarche at 7 years old with an increase in growth velocity (9 cm/year last 6 months) followed by premature reactivation of the hypothalamic-pituitary–gonadal axis, compatible with central precocious puberty (CPP). The LHRH stimulation test showed an LH peak of 10.5 U/L and FSH peak of 15.4 U/L with a LH/FSH relationship > 0.6. Her cousin was prenatally diagnosed with the deletion, who presented IUGR in the third trimester. At birth (40 weeks of gestational age), his weight was 2770 g (− 1.6SDS), length 46 cm (− 2.2SD) and showed muscular hypotonia. At 20 months old, he presented clinical traits of Temple as muscular hypotonia, hyperextensible joints, and facial traits (broad and prominent forehead, short nose with flat nasal root and wide tip, downturned corners of mouth, ears large and posteriorly rotated and micrognathia) and small hands.

**Table 1:** Clinical traits present in patients with Temple syndrome and in patients with small deletions reported

TS14 features	Present case	Dauber A [23] *n *= 4 (family)	Kagami M [2] *n* = 2 (family) *n* = 1		Sabria-Back [24] *n* = 1	TS14 features revised in Ioannides [3] and Kagami [18] series n = 83 patients	
Size of deletion	69 kb	14 kb	108 kb	411 kb	108 KB	1 Mb to 6 Mb	
Genetic etiology	*DLK1* whole-gene deletion	exon 1 *DLK1* deletion	*DLK1*, MEG3/DLK1:IG-DMR and MEG3:TSS-DMR deletion	*DLK1, RTL1*, MEG3/DLK1: IG-DMR and MEG3:TSS-DMR deletion	*DLK1*, MEG3/DLK1:IG-DMR and MEG3:TSS-DMR deletion	Deletion = 8	UPD = 63 Epimutation = 12
Sex Age	Female 4 years old	Females 6/8/6/7 years old	Female/Male 36/62 years old	Female 28 years old	Female NR (adulthood)	2 males:6 females	39 males:36 females
**Pregnancy and delivery**							
Premature birth	42 w	39–40 w	39w/NP	40w	NR	0% (0/6)	28% (18/65)
**Growth**							
Intrauterine growth retardation	-	NR	+/NR	+	NR	80% (4/5)	79% (49/62)
Low birth weight SDS ≤ -2.0	-1.6	-0.52/1.37/-0.07/0.98	-2.5/NR	− 2.2	NR	100% (4/4)	92% (58/63)
Short stature SD ≤ -2.0	-2.0*	0.6/-0.6/-0.6/0.7**	-2.2/-2.9	− 4.4	+	100% (8/8)	86% (62/72)
Obesity (BMI SDS)	Too Young (-0.8–0.6)	2/4 (2, > 2)**	-/ +	−	NR	20% (1/5)	41% (21/51)
**Head and neck**							
Relative macrocephaly at birth	+	-	NR/NR	+	NR	NR	52% (14/27)
Frontal bossing/Prominent forehead	+	-	+ / +	+	NR	50% (1/2)	64% (18/28)
High palate	−	-	NR/NR	−	NR	0% (0/1)	54% (14/26)
**Skeletal**							
Hyperextensible joints	+	NR	-/NR	−	NR	0% (0/3)	48% (22/46)
Small hands (percentile)	+ (p < 3)	−	+ / +	+	NR	100% (3/3)	86% (31/36)
Small feet	+	−	+ / +	+	NR	100% (1/1)	96% (25/26)
Clinodactyly	−	NR	-/NR	−	NR	0% (0/2)	42% (11/26)
**Central Nervous System**							
Hypotonia	+	-	-/NR	+	NR	80% (4/5)	82% (55/67)
Motor development delay	+	-	NR/NR	NR	NR	100% (3/3)	82% (31/38)
Speech delay	+	-	-/-	−	NR	100% (2/2)	56% (14/25)
Feeding problems	−	-	-/NR	−	NR	50% (1/2)	64% (18/28)
Mild Intellectual disability	+	-	-/-	−	NR	25% (1/4)	34% (14/41)
**Endocrine features**							
Early onset puberty	Precocious thelarche (7 years old)	4/4	+ /NR	-	+	60% (3/5)	70,6% (29/34)

W: weeks of gestation, NR: not reported. *at 2 years old;** adult

**Fig. 2:**
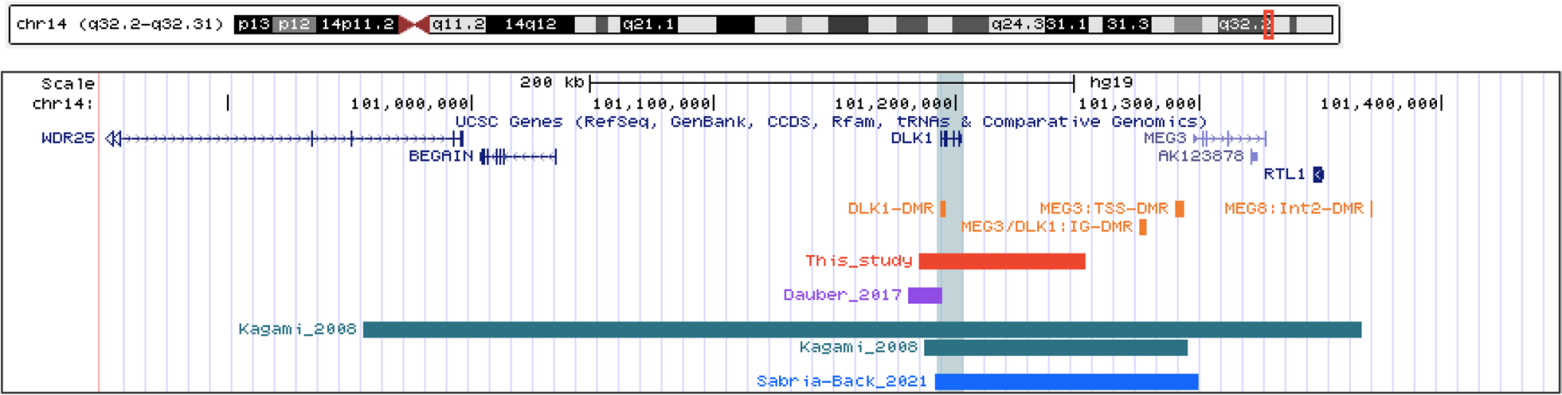
Deletions involving the *DLK1* gene identified in this study and those reported in the literature

**Fig. 3:**
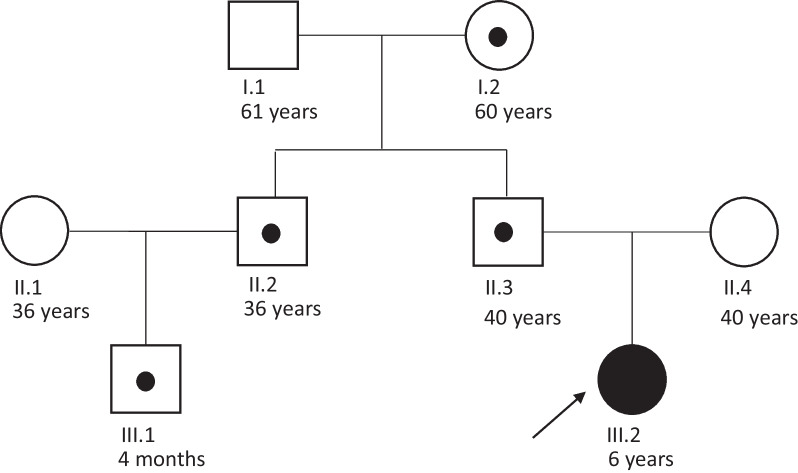
Family tree. III.2 is the TS14 index case. The remaining family members have normal phenotypes, with I.2, II.2, II.3 carrying the *DLK1* microdeletion on the maternal allele

Copy-number variation was assessed using aCGH. This revealed a normal female profile with an interstitial microdeletion at chromosome chr14q32.2 (arr[GRCh37] 14q32.2(101184595_101253432) × 1) with an approximate length of 69 Kb (Fig. 2). This deletion included the *DLK1* gene, consistent with the suspicion of TS14. The deletion was detected in her father (II.3), uncle (II.2) and grandmother (I.2), all of them with normal phenotype and her cousin recently born was prenatally diagnosed with the deletion (Fig. 3) and born with a low birthweight consistent with early features of TS14. Subsequently, MS-MLPA was used to test the methylation status at the *MEG3* DMR which resulted in a normal methylation profile in the proband and all deletion carriers. It must be noted that the MLPA kit used (ME021) does not include probes within other imprinted DMR on chromosome 14.

**Fig. 4:**
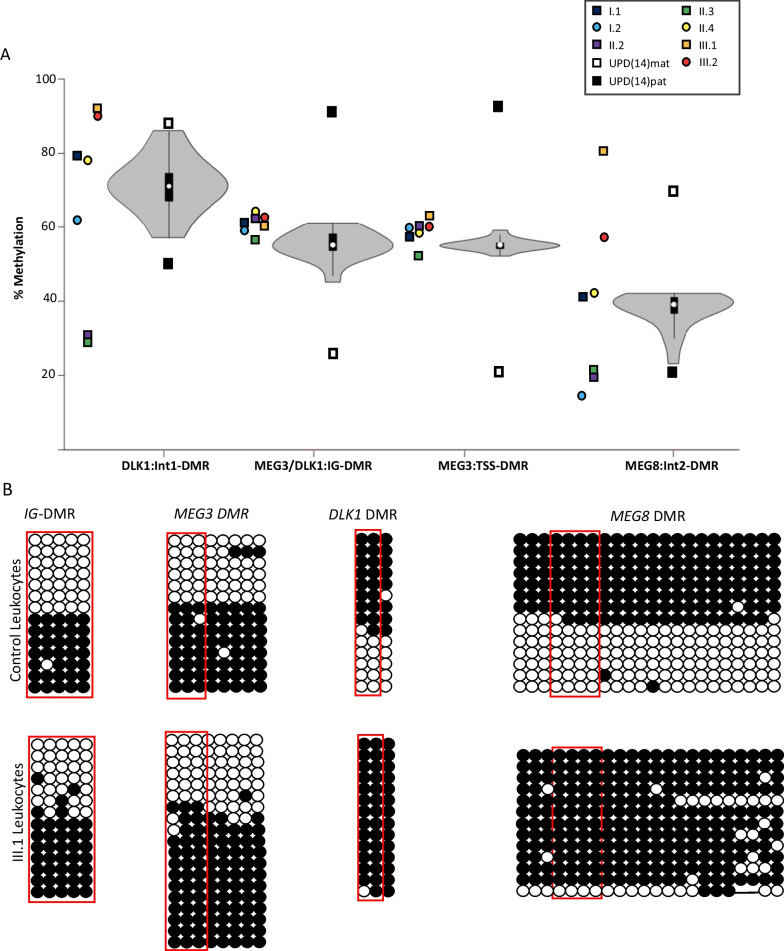
Methylation profiling of the TS14 index case and familial members. **A** Pyrosequencing was used to quantify methylation of CpG dinucleotides in the *DLK* DMR (maternally methylated), IG-DMR (paternally methylated), *MEG* DMR (paternally methylated) and the *MEG8* DMR (maternally methylated). Violin plots represent the average methylation profiles of 19 control individuals, with data points for family members located alongside. The black and white boxes above and below the violin plots represent methylation values for UPD(14)pat and UPD(14)mat control samples respectively. **B** Bisulfite PCR followed sub-cloning for amplicons from proband III.1 and a control leukocyte DNA sample. Each circle represent a single dinucleotide on the DNA stand. (•) methylated cytosine, (o) unmethylated cytosine. Each row corresponds to an individual cloned sequence. CpG dinucleotides in red box indicated those quantified by pyrosequencing

Extending characterization of allelic methylation throughout chr14q32 was performed in the index case (III.2), cousin (III.1), carriers (I.2, II.2, II.3) and non-deleted (II.4, I.1) family members using bisulfite PCR followed by pyrosequencing. This revealed IG-DMR and *MEG3* DMR profiles comparable with 19 healthy control samples, in agreement to MS-MLPA methylation observations, indicating allelic methylation was maintained correctly at these regions in the presence of the deletion (Fig. 4a; Additional file 1: Supplementary Table 1). The index patient showed relative hypermethylation at the *DLK1* DMR consistent with *DLK1* deletion on the paternal allele. Carrier members (II.2 and II.3) were relatively hypomethylated at this interval consistent with maternal transmission. Interestingly, the *MEG8* DMR was relatively hypomethylated (II.2 and II.3) in a similar way as the *DLK1* DMR, despite not being within the deleted interval (Fig. 4a). These methylation profiles were confirmed in the cloned PCR products (Fig. 4b). The grandmother (I.2) presented with discordant methylation profiles at these two maternally methylated intervals, with hypomethylation at the *MEG8* DMR but methylation within the normal range at the *DLK1* DMR. Pyrosequencing in the grandmother was independently replicated with similar profiles. It remains to be determined if methylation at these two somatically acquired maternally methylated intervals are mechanistically linked. Profiles obtained from the healthy control samples were subject to large inter-individual variation which may be attributed to the fact that allelic methylation at these two DMRs is not absolute and that the maternal allele is more methylated than the paternal allele [8, 9], plots and the range of methylation as defined by the UPD(14)pat and UPD(14)mat control samples (Fig. 4a). Furthermore, the normal unmethylated paternal allele at the *DLK1* DMR is associated with residual methylation, as indicated by the ~ 72% average methylation in the control cohort. Therefore, the normal methylation at the *DLK1* DMR in the grandmother, despite being at the lower end of the control range, may reflect variability which may become more evident with age.

On the basis of the presence of the microdeletion on the paternal allele and concomitant hypermethylation at the *DLK1* DMR, we confirmed the diagnosis of TS14 in the index case, despite normal methylation level at the *MEG3* and IG-DMR which are used for routine molecular diagnosis of this rare imprinting disorder.

## Discussion

Temple syndrome can result from different molecular anomalies. The majority of cases described have a UPD(14)mat, while imprinting defects and deletions are much rarer [3, 18]. Clinical findings are grossly similar among patients with UPD(14)mat, epimutations and deletions and are present in more than 60% of patients with TS14 [3, 18]. Phenotypic comparison among the few cases with deletion showed two phenotypes. Common features of TS14 are observed in *DLK1* whole deletions including short stature, small hands and feet, relative macrocephaly at birth, prominent forehead in infancy, hypotonia and CPP with motor development and intellectual disability. However, deletions restricted to the first exon of *DLK1* result in CCP only [23].

**Fig. 5:**
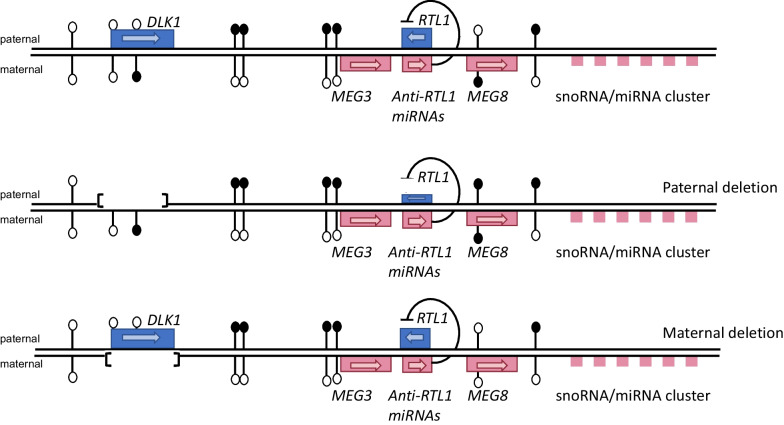
Structure of the imprinted 14q32 region, paternal and maternal *DLK1* gene deletion. Maternally expressed genes are indicated in red boxes and paternally expressed genes are indicated by blue boxes. The methylation is indicated by black lollipops. The *DLK1-DMR* is maternally methylated (Mat), *MEG3/DLK1:*IG-DMR and MEG3:TSS-DMR are paternally methylated (Pat) and *MEG8: Int2-DMR* is maternally methylated (Mat)

We have identified a child of healthy non-consanguineous parents with a phenotype of TS14 carrying a small deletion on 14q32 encompassing the *DLK1* gene with hypermethylation at the *DLK1* and *MEG8* DMR intervals and normal methylation at IG-DMR and *MEG3* DMR (Fig. 5). So far only eight patients with small deletions encompassing *DLK1* have been reported in the scientific literature (Fig. 2). Following Dauber et al. [23] description, rare small intragenic deletion in *DLK1* with deficiency of DLK1 measured in serum has been recognized as a genetic cause of familial CPP when paternally inherited.

The application of MS-MLPA in the affected individuals did not detect any additional copy number variation or abnormal methylation. Kagami et al. [2] described two families with three adult patients that were ascertained through familial studies following the birth of children with Kagami-Ogata syndrome (KOS14) in whom paternally transmitted *DLK1* deletions were identified. The first family (one male and one female) had a deletion of 108 Kb from *DLK1* to exon three of *MEG3*, including IG-DMR and *MEG3* DMR (https://www.ncbi.nlm.nih.gov/clinvar/variation/162007/). The affected patients did not demonstrate additional clinical TS14 features, except for a high prevalence of metabolic abnormalities, such as overweight/obesity and insulin resistance. In the second family, a female had a longer 411 Kb deletion containing *WD25, BEGAIN, DLK1, MEG3, RTL1, MEG8* including IG-DMR and *MEG3* DMR (https://www.ncbi.nlm.nih.gov/ clinvar/variation/ 162,009/). This case had pre-natal growth failure, severe short stature, frontal bossing, relative macrocephaly and small hands and feet. Neither of them exhibits motor development delay nor speech delay, clinical findings that are present in our patient and are present in more than 60% of TS14 cases. The final TS14 case was associated with a different 108 Kb deletion that removed the *DLK1* gene and both the paternally methylated DMRs. This individual was also found following intensive molecular analysis following the birth of a child with KOS14. This patient was initially undiagnosed, but had a constellation of TS14 phenotypic features including short stature, CPP and diabetes [24].

The index case described in this report had CPP as well as short stature, two main characteristics of TS14. Furthermore, she presented with many other clinical features of TS14 present in ≥ 50% of cases, such as relative macrocephaly, prominent forehead, small hands and feet, hypotonia, motor development delay and speech delay but contrary to the clinical review of Ioannides et al. [3] and Kagami et al. [18] she did not show feeding difficulties, IUGR, low birth weight, even though the birth weight was in p7 (− 1.5SD). Moreover, she showed hyperextensible joints, almond-shaped eyes, broad nasal tip and ears with attached lobe described in a lower frequency in TS14 [3, 18]. At present, a male cousin prenatally diagnosed with the 14q32 deletion who presented IUGR, at 20 months showed TS14 phenotype with prominent forehead, hyperextensible joints, small hands, and muscular hypotonia already present at birth. Together this suggests that lack of other paternally expressed genes, such as *RTL1* (or its epigenetic silencing) may be primarily responsible for the perinatal growth phenotypes as observed in the knock-out mouse model [25].

Rare heterozygous mutations in *DLK1* have also recently been recognized as a genetic cause of familiar CPP when paternally inherited. Indeed, three frameshift mutations in exon 5 of *DLK1* (p.Gly199Alafs*11, p.Val271Cysfs*14, and p.Pro160Leufs*50) were described in four women from three families with CPP. The segregation analysis was consistent with the maternal silencing of *DLK1* [26]. The affected patients did not demonstrate additional clinical TS14 features. Montenegro et al. [27] described a de novo heterozygous indel (c.401_404 + 8del) affecting exon 4 of *DLK1* gene with CPP. Importantly, the 3 mentioned studies that identified *DLK1* loss-of-function mutations as a monogenic cause of CPP (23,26,27) found undetectable serum levels of DLK1 in all affected individuals compared to controls. At present, patients with TS14 phenotype have not been described to carry *DLK1* point mutations. Nevertheless, five very rare cases with loss-of-function variants in *DLK1* are described in the GnomAD database and it is unknown whether the carriers of these variants who are reported in the database have any phenotypic abnormality.

The smallest overlapping region in patients with recognizable TS14 phenotype, based on the present case and those described above [18, 24], contains *DLK1* as the solely disrupted gene. The loss of function of the paternal copy of *DLK1* caused by whole-gene deletion, intragenic deletions, or frameshift mutation could lead to variable clinical features such as early pubertal signs (4.6–7 years) and additional TS14 associated features. *DLK1* encodes a transmembrane protein that is important for adipose tissue homeostasis and neurogenesis [28]. The exact mechanism by which *DLK1* regulates pubertal timing is not yet understood; however, it has been demonstrated in mouse that *Dlk1* is essential for neuroendocrine control of GnRh and the timing of puberty onset, in part through kisspeptin neurons [29]. *DLK1* could play a role in the regulation of neurogenesis within the hypothalamus, indirectly interfering with kisspeptin neuron formation, maturation, and/or secretion of kisspeptin through the activation or inhibition of Notch target genes. The disruption of the Notch signalling pathway, involved in neurogenesis and development of the hypothalamus, particularly in the reproductive axis, could be associated with central precocious puberty [28]. Moreover, *DLK1* has a wide fetal expression but in postnatal life, the expression decreases, except in endocrine glands, mainly in adrenals, ovaries, adipose tissue, pituitary, and hypothalamic nuclei. These data seemed to be consistent with the pubertal and metabolic phenotypes presented by patients described with DLK1 deficiency. Recently, despite Dlk1-null mice displayed fetal growth restriction, in humans, *DLK1* variants were not associated with being born small for gestational age in a large cohort of patients with pre- and postnatal growth failure (Silver-Russell phenotype) [30].

Psychomotor delay, mild intellectual disability and language delay present in our patient and reported in all the molecular etiologies of TS14 could be indirectly regulated by *DLK1*. Using the STRING tool (Search tool for Retrieval of Interacting Genes / Proteins) (https://string-db.org/) to obtain information about molecular networks and removing text mining with a high confidence score (> 0.7) revealed that *DLK1* is connected with *NOTCH1* and *FIBP* genes reporting confidence scores of 0.93 and 0.75 respectively. These genes are considered as intellectual disability candidate genes in the SysID database with mild or limit intellectual functioning. Pathogenic variants in *NOTCH1* are associated with Adams-Oliver syndrome, a third of whom have intellectual disability [31] and loss-of-function mutation in the *FIBP* gene underlies an autosomal recessive syndromic overgrowth (mainly in height) associated with macrosomia, learning disabilities/developmental delay, and other congenital defects [32]. In addition, DLK1 is necessary for hippocampal neurogenesis in the regulation of neural stem cells maintenance in the long term modulation and for normal cognitive functions such as spatial memory consolidation [33].

Detailed methylation analysis by pyrosequencing confirmed the results obtained by MS-MLPA with normal methylation levels at the IG-DMR and *MEG3* DMR. Normal methylation at these paternally methylated DMRs has not been reported in TS14 patients previously [14, 34], including patients with large and small deletions of the 14q32 region. Patients with paternal deletions of *DLK1-MEG3*, UPD(14)mat and epimutations all possess hypomethylation at the IG-DMR and *MEG3* DMR. The relative hypermethylation at *DLK1* DMR we observed is consistent with the paternally inherited deletion eliminating the unmethylated paternal allele of *DLK1*. Furthermore, the relative hypomethylation at the *DLK1* DMR in the proband’s father and uncle is consistent with maternal transmission.

Interestingly, the *MEG8* DMR also showed a reciprocal methylation pattern depending on transmission similar to that observed at the *DLK1* DMR, despite not being deleted. The relative hypomethylation of these two somatically acquired DMRs suggests that methylation of *MEG8* is dependent on, or secondary to the appropriate methylation at the *DLK1* DMR. Such hierarchical dynamics have been described previously for the DMRs within the *Igf2-H19* domain [28]. Recent targeted manipulation of allelic methylation within the orthologous domain in mouse using the dCas9-fusion system has revealed complex interactions between DMRs [35]. Although DNA methylation at the *Dlk1* DMR was not directly targeted, dCas9GCN4-scFv-TETCD mediated IG-DMR hypomethylated resulted in down-regulation of both *Dlk1* and *Rtl1* and *Meg8* DMR hypermethylation on the paternal allele. Together this suggests altered methylation at the IG-DMR causes domain-wide epigenotype switching, endorsing the existence of hierarchical allelic methylation similar to what we report in this family.

The function of the *DLK1* DMR is unknown, but it is unlikely to directly influence the monoallelic expression since it is methylated on the expressed allele; however, this may reflect gene body methylation associated with silencing of cryptic transcript initiation. Recently, the *Meg8* DMR was deleted in a mouse cell line model [36] and embryos [37]. This revealed that removal of the DMR, or only a CTCF insulator site, in MLTC-1 cells resulted in significant reduction in the paternally expressed *Dlk1* and *Rtl1* genes, and upregulation of the *Meg3* and *Rian*. Similar results were observed in knock-out mice, with the exception that *Dlk1* was not altered. The resulting knock-out did not grossly affect embryonic development [37]. Analysis of allelic methylation revealed that *Meg8* DMR deletion did not result in changes within the cluster. Therefore, methylation at the IG-DMR, *Meg3* DMR or *Dlk1* DMR may influence *Meg8* DMR establishment, but the reciprocal is not true, implying that *Meg8* is the last DMR to be established, which is supported by the methylation profile in our proband. Interestingly, in humans, the *MEG8* DMR is located only 4 Kb away of the *RTL1* leader exon (NM_001134888), suggesting the *MEG8* DMR may function as a cis-regulatory element. Interrogation of ENCODE ChIP-seq data revealed conserved CTCF binding within the *MEG8* DMR, consistent with this maternally methylated interval contributing to allele-specific chromatin confirmation throughout the domain.

Irrespective of the molecular mechanism, individuals with TS14 generally lack *DLK1* and *RTL1* expression and it is highly likely that both of these paternally expressed genes are related to the growth, muscular and behavioral phenotypes reported. Despite the fact paternal *RTL1* is intact in our proband, expression is likely reduced due to hypermethylation of the *MEG8* DMR. Unfortunately, due to the tissue restricted expression of *RTL1*, we could not test this hypothesis in the proband.

## Conclusion

Here, we described a small 14q32 paternal microdeletion encompassing the whole *DLK1* gene in a patient with phenotypes resembling TS14. This patient has normal methylation at the paternally methylated imprinting control regions, IG-DMR and *MEG3* DMR; therefore, normal DNA methylation testing of *MEG3* DMR was inconclusive for TS14 in this case. Our results suggested that the maternally methylated DMRs located within the *DLK1* and *MEG8* genes could be also tested in patients with suspected TS14 to determine relative hypermethylation that could result from paternal microdeletions of *DLK1*.

### Methods

## Microarray

An exon array comparative genomic hybridization (aCGH) 4 × 180 K ISCA (Oxford Gene Technology, OGT) was carried out for genomic-wide copy number analysis following the manufacturer’s protocol. Data analysis was performed with Cytosure software.

## Methylation-specific multiplex ligation-dependent probe amplification (MS-MLPA)

Gene dosage and methylation analyses of the 14q32 chromosomal region, including the *MEG3* DMR, were carried out using the SALSA MLPA Kit ME032-A1 (MRC Holland, Amsterdam, The Netherlands) according to the manufacturer’s manual. Amplification products were run on an ABI3130 Genetic Analyzer (Applied Biosystems, California, USA) and analyzed using the GeneMapper software (Applied Biosystems).

## Bisulfite PCR and pyrosequencing

Five hundred nanograms of genomic DNA were subjected to sodium bisulfite treatment and purified using the EZ GOLD methylation kit (ZYMO) following the manufacturer’s recommendations. DNAs from 19 normal controls were utilized to define the normal range of methylation while patients carrying UPD(14)pat and UPD(14)mat were used to detect extreme methylation values at the imprinted DMRs assessed. Approximately 50 ng of bisulfite converted DNA was used as template for PCR amplification of the *MEG8* DMR, *DLK1* DMR, IG-DMR and *MEG3* DMR using Immolase Taq polymerase (Bioline, London, UK) at 45 cycles in which one primer was biotinylated (details of primer sequences available in Sabria-Back et al. [24]). One ul of each PCR productions from proband III.1 was cloned into pGEMT-easy vector (Promoga) for strand-specific methylation analysis, and the remainder was used in the pyrosequencing reaction. The entire biotinylated PCR product (diluted to 40 μl) was mixed with 38 μl of binding buffer and 2 μl (10 mg/ml) streptavidin-coated polystyrene beads. After incubation at 65◦C, DNA was denaturated with 50 μl 0.5MNaOH. The single-stranded DNA was hybridized to 40-pmol sequencing primers dissolved in 11 μl annealing buffer at 90◦C. For sequencing, a primer was designed on the opposite strand to the biotinylated primer used in the PCR reaction. The pyrosequencing reaction was carried out on a PyroMark Q24 instrument (Qiagen, Hilden, Germany). The peak heights were determined using Pyro Q-CpG1.0.9 software (Qiagen).

### Supplementary Information

**Additional file1**: Supplementary table 1. Pyrosequencing results for the affected family and controls.

## Data Availability

Data are available from the corresponding author on request.

## Abbreviations

TS14: Temple syndrome
DMR: Differentially methylated regions
UPD(14)mat: Maternal uniparental disomy 14
PWS: Prader-Willi syndrome
SRS: Silver-Russell syndrome
SD: Standard deviation
aCGH: Array comparative genomic hybridization
MS-MLPA: Methylation-specific multiplex ligation-dependent probe amplification
KOS14: Kagami-Ogata syndrome
CpG: Cytosine-guanine dinucleotide
